# Rare incidence of methotrexate-specific lesions in liver biopsy of patients with arthritis and elevated liver enzymes

**DOI:** 10.1186/ar3085

**Published:** 2010-07-16

**Authors:** Emilie Quintin, Jean-Yves Scoazec, Hubert Marotte, Pierre Miossec

**Affiliations:** 1 Clinical Immunology Unit, Departments of Immunology and Rheumatology, Edouard Herriot hospital, Place d'Arsonval, 69437 Lyon, France; 2 Department of Pathology, Edouard Herriot hospital, Place d'Arsonval, 69437, Lyon, France

## Abstract

**Introduction:**

The present study objective was to evaluate the incidence of methotrexate (MTX)-specific liver lesions from the analysis of a liver biopsy of inflammatory arthritis patients with elevated liver enzymes.

**Methods:**

A case-control study was performed with 1,571 arthritis patients on long-term low-dose MTX therapy. Results of liver biopsy were analyzed in 41 patients with elevated liver enzymes. The expression of autoimmune markers was also assessed. This population was compared with 41 disease control subjects obtained from the same database, also on MTX but without elevated liver enzymes, matched for age, sex and rheumatic disease.

**Results:**

Compared with the disease controls, patients with liver biopsy showed lower disease duration and lower MTX exposure, weekly and cumulative doses, reflecting shorter treatment duration due to liver abnormalities. Liver biopsies showed 17 autoimmune hepatitis-like (AIH-like) lesions, 13 nonalcoholic steatohepatitis-like lesions, seven limited liver lesions, and two primary biliary cirrhoses. However, MTX-specific lesions with dystrophic nuclei in hepatocytes were seen in only two cases. Liver biopsy lesions were associated with autoimmune markers (*P *= 0.007); notably, AIH-like lesions were associated with rheumatoid arthritis and with the presence of the HLA-DR shared epitope.

**Conclusions:**

MTX-specific liver lesions are rarely observed in arthritis patients under long-term MTX therapy and elevated liver enzymes.

## Introduction

Low-dose methotrexate (MTX) has been used for the treatment of inflammatory disorders since 1950. The early observation that psoriatic arthritis responded to MTX was followed by clinical trials in other inflammatory diseases such as rheumatoid arthritis (RA), primary Sjögren syndrome (pSS) and connective tissue diseases (CTD) with a rather good safety profile [1]. Today, low-dose MTX therapy is the most commonly used treatment for rheumatic diseases [2]. Weekly low-dose MTX treatment acts primarily as an anti-inflammatory drug, specifically through the release of adenosine, rather than as an anti-metabolite drug as in cancer [3,4]. Even at low doses, MTX has been associated with hepatic disorders, mostly elevated liver enzymes that may lead to treatment cessation [5-7]. This association has led to the publication of guidelines for the clinician [5,8,9].

In inflammatory arthritis patients on long-term low-dose MTX, the contribution of the underlying rheumatic disease has been incriminated in the genesis of these liver lesions, with MTX acting possibly as a cofactor [10,11]; however, this is not fully demonstrated. A large variety of histological liver lesions has been described, including dystrophic nuclei, macrovesicular steatosis, cell necrosis, cholestasis, Ito cell hyperplasia, portal inflammation, liver fibrosis [12-14] and even cirrhosis [15-17]. The severity of these lesions has been associated with the duration of treatment in some conditions [13,18]. Importantly, many of these lesions are observed in the absence of MTX. Some cell changes with dystrophic nuclei in hepatocytes, however, are considered the most MTX-specific lesions, but their real incidence is not really known [15].

The present report aimed to assess the incidence of the most MTX-specific lesions [15] and the contribution of the underlying rheumatic disease in the development of hepatic disorders in a large cohort of arthritis patients on long-term low-dose MTX. Histological characteristics of liver biopsies and biological autoimmune markers from 41 arthritis patients with elevated liver enzymes while on MTX treatment were analyzed.

## Materials and methods

### Patient inclusion criteria

Among 2,492 patients followed for chronic inflammatory arthritis in our unit, a population of 1,571 patients on long-term low-dose MTX therapy was studied. All details regarding clinical features, treatment dosing and laboratory follow-up were available from a single computer database. Liver enzymes were followed every month in all patients as part of their routine follow-up. Forty-one patients with an increase of liver enzymes (transaminases >3 upper limits of normal) for more than 3 weeks after stopping MTX underwent a liver biopsy. Transaminases were still elevated at the time of biopsy. None had liver enzyme elevation at baseline prior to starting MTX treatment. No other obvious liver risk factor was reported (alcohol consumption, obesity, other obvious hepatotoxic drugs), except diabetes in three patients. Patients positive for hepatitis B and hepatitis C were excluded. For each subject, age, disease duration, MTX exposure duration, MTX weekly doses and MTX cumulative doses were recorded, as well as biological markers linked to autoimmunity - including rheumatoid factor, anti-cyclic citrullinated peptide (anti-CCP) antibodies, antinuclear antibodies, anti-DNA antibodies, anti-smooth muscle antibodies (SMA), liver kidney microsomal type 1 (LKM1) antibodies, anti-mitochondrial antibodies, HLA-DR shared epitope (HLA-DRB1*0401, HLA-DRB1*0404, HLA-DRB1*0405, HLA-DRB1*0408, HLA-DRB1*0101, HLA-DRB1*0102, HLA-DRB1*1001, HLA-DRB1*1402), HLA-B27 and HLA-DRB1*03.

To compare the populations with and without liver disease, the patients with liver biopsy were compared with a population of 41 arthritis patients obtained from the same database of 1,571 patients also on MTX therapy but without elevated liver enzymes on their monthly follow-up, exactly matched for age, sex, and type of inflammatory disease. The protocol was approved by the ethical committee for clinical research of the University Hospitals of Lyon and of the French Ministries of Health and of Research, and all patients gave their informed written consent.

Patients and disease controls were split into four groups according to the main associated chronic inflammatory arthritis: RA, according to the American College of Rheumatology criteria [19]; spondylarthropathy (SpA), according to the European Spondylarthropathy Study Group criteria, including ankylosing spondylitis and psoriatic arthritis [20]; pSS, according to the European revised criteria [21]; and CTD, including systemic lupus erythematosus and mixed CTD.

### Analysis of liver biopsy

The analysis focused on the histological findings of an echo-guided liver biopsy performed in 41 patients. No immediate or secondary event was noticed during and after liver biopsy. Samples were of good quality (>15 mm long) and were fixed in buffered formalin and embedded in paraffin. Four-micrometer tissue sections were stained with hematoxylin and eosin, chromotrope, Masson's trichrome and Perls' staining. All liver biopsies were analyzed by the same pathologist (J-YS), unaware of the clinical details other than the inclusion in the protocol.

The following parameters were studied: portal inflammation, graded from 0 to 3; lobular inflammation, graded from 0 to 3; composition of the inflammatory infiltrates, especially the presence of plasma cells and/or eosinophils; presence, extent and distribution of hepatocellular necrosis; presence, extent and distribution of fibrosis as periportal, perisinusoidal and centrolobular; presence of dystrophic nuclei in hepatocytes, which has been previously linked to direct MTX liver toxicity [15]; presence, extent and distribution of steatosis; and other lesions (biliary alterations, vascular changes, and so forth).

### Statistical analysis

Analysis of variance was performed to test the effect of diagnostic group and type of biopsy finding on mean age, disease duration, MTX exposure, cumulative and weekly doses using the SygmaStat^® ^statistical analysis software (Systat software, Chicago, IL, USA). The significant analysis of variance results were adjusted by post-hoc Bonferroni *t *test. The chi-square test was carried out to test the association between groups and autoimmune marker expression. Statistical comparisons between patients and disease controls used the Student *t *test or the Mann-Whitney rank sum test when the test for normality failed. All values are presented as the mean ± standard error of the mean. *P *< 0.05 was considered significant.

## Results

### Patient population

Forty-one patients out of a total population of 1,571 arthritis patients on MTX treatment and with elevated liver enzymes underwent a liver biopsy (2.6%), and were compared with 41 disease control subjects without elevated liver enzyme, matched for age, sex, and inflammatory disease. For obvious practical and ethical reasons, the control population was not exposed to the potential risk of a liver biopsy.

Statistical comparisons showed no difference between the two groups, except for lower mean values for disease duration (*P *= 0.01), for MTX exposure duration (*P *< 0.001), and for MTX cumulative dose (*P *< 0.001) in the patients with liver biopsy compared with their disease controls (for details, see Table 1). These results reflect an earlier termination of MTX in biopsied patients because of hepatic toxicity.

**Table 1:** Characteristics of patients with liver biopsy and of disease control patients

	Patients with liver biopsy	Disease control patients	*P *values
Sex (female/male)	28/13	28/13	NS
Age (years)	52.2 ± 1.9	52.4 ± 1.9	NS
Rheumatic disease	25 RA; 8 SpA; 6 pSS; 2 CTD	25 RA; 8 SpA; 6 pSS; 2 CTD	NS
Disease duration (years)	7.4 ± 1.1	10.5 ± 1.1	0.01
MTX exposure (weeks)	131.6 ± 26	277.9 ± 29.6	<0.001
MTX cumulative dose (mg)	1,287.8 ± 246.7	3,217.3 ± 368	<0.001
MTX weekly dose (mg/week)	10.9 ± 0.7	12.240 ± 0.7	NS
Shared epitope positivity	17/36	22/41	NS
Rheumatoid factor positivity	17/38	19/39	NS
Anti-CCP positivity	19/29	12/23	NS

Data for 41 patients with liver biopsy compared with 41 subjects matched for age, sex and rheumatic disease from a population of 1,571 arthritis patients on long-term methotrexate (MTX) therapy. Data presented as *n *or mean ± standard error of the mean. CCP, cyclic citrullinated peptide; CTD, connective tissue disease; NS, not significant; pSS, primary Sjögren syndrome; RA, rheumatoid arthritis; SpA, spondylarthropathy.

When considering the population of 41 patients with liver biopsy, the distribution of the rheumatic diseases was as follows: 25 RA cases, eight SpA cases (including four ankylosing spondylitis and four psoriatic arthritis), six pSS cases and two CTD cases (including one systemic lupus erythematosus and one mixed CTD). These groups of patients significantly differed only by their mean age (*P *= 0.01) but not by either disease duration, MTX exposure duration, or cumulative or weekly dose mean values (data not shown). There was an expected link between the rheumatic disease and the expression of autoimmune markers. The presence of rheumatoid factor, anti-CCP antibodies and the HLA-DR shared epitope was mostly reported with RA patients. No significant association was observed between the rheumatic disease and the other autoimmune markers such as antinuclear antibodies, anti-DNA antibodies, and presence of HLA-B27 or HLA-DRB1*03.

### Histological findings of liver biopsies

The histological findings of liver biopsies showed a spectrum of lesions, which could for clarity be divided into five groups (Table 2). The most common liver lesions were autoimmune hepatitis-like (AIH-like) lesions, found in 17/41 patients (41.5%). These lesions were characterized by the presence of portal and/or lobular inflammatory infiltrates, usually rich in plasma cells, associated with hepatocellular necrosis (piecemeal necrosis or intralobular necrosis). These lesions were usually mild and the necrotic-inflammatory activity was mild to moderate, and only a limited amount of fibrosis was usually present. If present, steatosis was usually mild. There was no evidence of dystrophic nuclei to suggest a direct MTX toxicity [15].

**Table 2:** Distribution of the histological liver lesions depending on the associated rheumatic disease

	AIH-like lesions	NASH-like lesions	Limited liver lesions	PBC	Direct MTX toxicity	Total
Rheumatoid arthritis	13 (31.7%)	9 (22%)	1 (2.4%)	1 (2.4%)	1 (2.4%)	25 (61%)
Spondylarthropathy	2 (4.9%)	3 (7.3%)	2 (4.9%)	0 (0%)	1 (2.4%)	8 (19.5%)
Primary Sjögren syndrome	0 (0%)	1 (2.4%)	4 (9.8%)	1 (2.4%)	0 (0%)	6 (14.6%)
Connective tissue disease	2 (4.9%)	0 (0%)	0 (0%)	0 (0%)	0 (0%)	2 (4.9%)
	17 (41.5%)	13 (31.7%)	7 (17%)	2 (4.9%)	2 (4.9%)	41 (100%)

Data presented as *n *(%). AIH, autoimmune hepatitis; MTX, methotrexate; NASH, nonalcoholic steatohepatitis; PBC, primary biliary cirrhosis.

The second most common liver lesions were nonalcoholic steatohepatitis (NASH) lesions found in 13/41 patients (31.7%). These lesions were characterized by marked macrovesicular steatosis (in more than 30% of hepatocytes), glycogen-laden nuclei and hepatocellular ballooning. Minimal inflammation might be present. Fibrosis, when present, was mainly perisinusoidal and predominated into the centrilobular areas.

The third pattern was limited liver lesions, reported in 7/41 patients (17%), consisting of mild portal or lobular inflammatory infiltrates, with no or limited evidence of hepatocellular fibrosis. The overall histological picture was poorly specific and the lesions were not clear enough to be classified as part of another group.

In two cases (4.9%), primary biliary cirrhosis (PBC) fulfilling all histological criteria [22] was discovered in these PBC-asymptomatic patients. Both cases were negative for antimitochondrial antibodies. These lesions were detected only because the patients were on MTX with monthly liver enzyme determinations.

Finally, lesions directly related to the hepatic toxic effects of MTX were reported in only two cases (4.9%). The lesions combined steatosis, hepatocytes with dystrophic nuclei and extensive perisinusoidal fibrosis, with no or very limited inflammation [15]. The specific contribution of the drug, despite a similar exposure to MTX in the 41 patients, could therefore be demonstrated in a very limited number of cases.

It is to be noted that nonextensive lesions of fibrosis were observed in 11 out of 17 AIH-like lesions (65%), in eight out of 13 NASH-like lesions (61.5%) and in the two cases of PBC, and were not observed in limited liver lesions (*P *= 0.01).

### Associations between histological lesions and biological markers

As shown in Table 3, the MTX exposure duration and cumulative or weekly doses did not differ between groups of histological lesions. On the other hand, there was a link between the histological lesions and the underlying rheumatic disease (*P *= 0.04). AIH-like lesions were mostly seen in RA patients (13 out of 25 RA patients; 52.5%), in two out of eight patients with SpA (25%), in two out of two patients with CTD and not in patients with pSS. NASH-like lesions were observed in nine out of 25 RA patients (36%), in three out of eight patients with SpA (37.5%), in one out of six patients with pSS (16.7%) and not in patients with CTD. Limited liver lesions were found in one out of 25 RA patients (4%), in two out of eight patients with SpA (25%), in four out of six patients in pSS (66.7%) and not in patients with CTD. PBC was observed in one out of 25 RA patients (4%), in one out of six patients with pSS (16.7%) and not in patients with SpA or CTD. Liver lesions related to direct MTX toxicity were found in only two cases, one in RA and the other in SpA (for details, see Table 2).

**Table 3:** Characteristics of the patients with liver biopsy depending on the histological liver lesions

	AIH-like lesions (*n *= 17)	NASH-like lesions (*n *= 13)	LL lesions (*n *= 7)	PBC (*n *= 2)	Direct MTX toxicity (*n *= 2)	*P *value
Sex (female/male)	13/4	8/5	5/2	2/0	0/2	NS
Age (years)	55.2 ± 3.2	51.2 ± 3.2	47.7 ± 4.5	57.5 ± 6.5	44.5 ± 1.5	NS
Disease duration (years)	10.5 ± 2.2	5.2 ± 1.1	4.6 ± 1.8	6.2 ± 1.8	6.9 ± 4.9	NS
MTX exposure (weeks)	139.9 ± 44.8	132.8 ± 33.8	26.1 ± 7.2	227.6 ± 158.2	325.7 ± 282.9	NS
MTX cumulative dose (mg)	1,260.9 ± 324.3	1,458.4 ± 460.9	306.1 ± 77	1,676.9 ± 1,156.9	3,454.2 ± 3,025.7	NS
MTX weekly dose (mg/week)	10.2 ± 1.7	12 ± 1.3	11.6 ± 1.6	7.4 ± 0.1	10.3 ± 0.3	NS
Patients on steroid	7/17	4/13	1/7	0/2	0/2	NS
Fibrosis	11/17	8/13	0/7	2/2	2/2	0.01
Shared epitope positivity	12/15	4/12	1/7	0/2	ND	0.01
HLA-B27 positivity	3/15	1/12	2/7	0/2	ND	NS
HLA-DRB1*03 positivity	1/15	2/12	2/7	1/2	ND	NS
Rheumatoid factor positivity	8/15	6/12	2/7	0/2	1/2	NS
Anti-CCP positivity	14/16	5/9	0/2	0/1	0/1	0.01
ANA positivity	10/16	4/13	2/7	2/2	1/2	NS
Anti-DNA antibody positivity	2/15	0/13	2/7	0/2	0/2	NS
Anti-SMA positivity	2/12	0/9	1/7	0/2	0/2	NS
Anti-LKM1, positivity	0/12	0/9	0/7	0/2	0/2	ND
AMA positivity	2/12	0/9	0/7	1/2	0/2	NS

Data presented as *n *or mean ± standard error of the mean. AIH, autoimmune hepatitis; AMA, antimitochondrial antibodies; ANA, antinuclear antibodies; CCP, cyclic citrullinated peptide; LKM1, liver/kidney microsomal antibodies type 1; LL, limited liver; MTX, methotrexate; NASH, nonalcoholic steatohepatitis; ND, not determined; NS, not significant; PBC, primary biliary cirrhosis; SMA, smooth muscle antibodies.

Furthermore, there was an association between the liver lesions and the shared epitope (*P *= 0.007). Given that the liver lesions were found to be associated with the underlying disease - specifically RA - and that RA is associated with the shared epitope (for details, see earlier Patient population), such an association was expected. The shared epitope was mostly found in patients with AIH-like lesions (12/15), and less in patients with NASH-like lesions (4/12). No significant association was observed between a particular histological lesion and the other autoimmune markers such as rheumatoid factor, anti-CCP, antinuclear antibodies, anti-DNA, anti-SMA, anti-mitochondrial antibodies, anti-LKM1 antibodies, HLA-B27 and HLA-DRB1*03 (Table 3). In addition, patients with AIH-like lesions were mostly negative for the antibodies commonly found in autoimmune hepatitis (anti-SMA or anti-LKM1 antibodies [23]), suggesting a particular entity.

## Discussion

An increase of liver enzymes is the most common adverse event observed in clinical practice in arthritis patients under long-term MTX therapy; however, the exact mechanism of such disorder remains unclear. Combining histological and immunological approaches, the present study indicated that the direct role of long-term low-dose MTX in the genesis of a MTX-specific liver change with dystrophic nuclei in hepatocytes was very rarely observed. In contrast, autoimmune lesions were commonly found.

Previous results have suggested a poor correlation between the level of increase of liver enzymes and histological findings on liver biopsies in arthritis patients under long-term MTX therapy [15-17,24,25]. In postmortem liver biopsies performed on 188 subjects with RA on long-term low-dose MTX therapy, Ruderman and colleagues reported only two cases of hepatic fibrosis, which were not directly related to MTX but related to either chronic alcoholism or chronic viral hepatitis [26]. Richard and colleagues performed systematic liver biopsies prior to the introduction of long-course low-dose MTX and after 1 year of therapy. Eleven cases of hepatic fibrosis were observed prior to the introduction of MTX, with no sign of worsening at 1 year [27]. More recently, no correlation was found between liver MTX concentrations and incidence of liver toxicity [28]. Based on these results, liver biopsy is no longer performed on a systematic basis after exposure to usually 2 g MTX.

In the present study, histological findings with dystrophic nuclei consistent with a direct MTX toxicity [15] were found in only two patients. These changes are not found with nonsteroidal anti-inflammatory drug therapy, the drugs most commonly found in association with MTX. It should be noticed that NASH-like lesions were not associated with fibrosis and dystrophic nuclei. As such, they were not classified as MTX-related because of the absence of the last two parameters. These isolated liver changes could result from a large number of causes, including systemic inflammation itself, metabolic abnormalities, namely diabetes, and alcohol intake. In the other changes, the heterogeneity of the histological lesions (that is, mainly AIH-like and NASH-like) and the fact that none of these appeared as MTX dose-dependent suggest a poor link between these liver lesions and MTX administration. This conclusion is in contrast with previous but older studies that concluded a need to perform sequential liver biopsies to detect direct MTX liver toxicity [18,29]. The current recommendations do not reach the same conclusion [8]. In our study, neither NASH-like lesions nor the other histological lesions appeared MTX dose-dependent, consistent with previous data [30,31]. These results are in favor of a weak direct liver toxicity of long-term MTX in arthritis patients. We reached the same conclusion on safety in patients with arthritis and hepatitis C treated with MTX or etanercept [32,33].

Interestingly, a link between the histological liver lesions and the underlying rheumatic disease was observed. The association between autoimmune rheumatic disease and hepatic lesions has been previously reported. In our study, AIH-like lesions were strongly associated with RA and with the presence of the shared epitope. An interesting and new observation is that the presence of the antibodies commonly found in AIH (anti-SMA and anti-LKM1 antibodies [23]) was mostly negative in these RA-associated AIH-like lesions. The reason for this observation is unclear but could be linked to the rather modest intensity of the lesions. On the other hand, anti-CCP antibodies, also described in type 1 autoimmune hepatitis, were observed in almost 90% of patients with AIH-like lesions.

Overall, the results of the present study lead one to reconsider the role of autoimmunity in the induction of hepatic disorders in arthritis patients under long-term low-dose MTX therapy. Indeed, in these patients, elevated liver enzymes were rarely the consequence of direct MTX toxic effects. The presence of an underlying latent liver disease, possibly autoimmune in nature, was commonly found - especially in RA patients. In this particular context, exact causality assessment in drug-related liver injury remains a difficult challenge, even using the new criteria [34]. To assess the real influence of autoimmune mechanisms in the genesis of hepatic disorders in arthritis patients on MTX treatment, it would be necessary to perform a prospective study with liver biopsy both before the introduction of MTX and during treatment in control patients without elevated liver enzymes. Today, such an approach is difficult - even impossible - to follow from an ethical point of view.

## Conclusions

MTX-specific liver lesions are rarely found in arthritis patients. An early increase of liver enzymes should not lead systematically to stopping MTX treatment. Liver biopsy remains of interest to diagnose independent or poorly related liver lesions. This technique may identify a latent autoimmune liver disease, possibly related to persistent activity of the inflammatory disease. Other modes of treatment including cytokine inhibitors could be indicated in this context.

## Abbreviations

AIH: autoimmune hepatitis; CCP: cyclic citrullinated peptide; CTD: connective tissue diseases; LKM1: liver/kidney microsomal type 1 antibodies; MTX: methotrexate; NASH: nonalcoholic steatohepatitis; PBC: primary biliary cirrhosis; PSS: primary Sjögren syndrome; RA: rheumatoid arthritis; SMA: smooth muscle antibodies; SPA: spondylarthropathy.

## Competing interests

The authors declare that they have no competing interests.

## Authors' contributions

EQ participated in the design of the study, collected the data, performed the statistical analysis, and drafted the manuscript. J-YS performed the analysis of the liver biopsies. HM contributed to the follow-up of patients and to data collection. PM participated in drafting and editing the manuscript and is responsible for this manuscript. All authors read and approved the final manuscript.
